# Coarsening Behavior of Particles in Fe-O-Al-Ca Melts

**DOI:** 10.1038/s41598-019-40110-x

**Published:** 2019-03-06

**Authors:** Linzhu Wang, Junqi Li, Shufeng Yang, Chaoyi Chen, Huixin Jin, Xiang Li

**Affiliations:** 1 School of Materials and Metallurgy, Guizhou University, Guiyang, Guizhou 550025 China; 2 School of Metallurgical and Ecological Engineering, University of Science and Technology Beijing, Beijing, 100083 China; 3 College of Materials & Metallurgical Engineering, Guizhou Institute of Technology, Guiyang, 550003 China

## Abstract

The characteristics of particles greatly affect the microstructure and performance of metallic materials, especially their sizes. To provide insight into coarsening phenomena of particles in metallic melts, Fe-O-Al-Ca melt with calcium aluminate particles was selected as a model system. This study uses HT-CSLM, SEM detections and stereological analysis to probe the behavior of particles and their characteristics including size, number density, volume fraction, spreading of particle size, inter-surface distance and distribution of particles. Based on the experimental evidence and calculation of collision, we demonstrate that the coarsening of inclusion particles is not only dependent on the Ostwald growth as studied in previous study, but also on the particle coagulation, and floatation. The collision of particles affects the maximum size of the particles during whole deoxidation process and dominates the coarsening of particles at later stage of deoxidation under the condition without external stirring in Fe-O-Al-Ca melts. The factors influencing collision behaviors and floating properties were also analyzed, which is corresponding to coarsening behavior and change of particle characteristic in the melts with different amounts of Ca addition. Such coarsening mechanism may also be useful in predicting the size of particles in other metallic materials.

## Introduction

Particles are inevitable products in metallic materials which form during metal refining, casting, and thermal processing in liquid or solid metals^1–4^. It has been a common knowledge that particles play a significant role in determining the continuous castability and performances of materials^5–7^. The Al_2_O_3_ particles with high hardness and high melting temperature generate during deoxidation process in Al-killed steels may lead to nozzle clogging^8^. Another possible side effect is a decrease in machinability and service life, caused by the nucleation and propagation of voids around precipitate particles on the weak grain boundaries^5,7,9^. However, the effect of particles on the properties of metal is significantly dependent on the particle size distribution, spatial distribution, morphology and composition of particles. Lots of scholars have verified that the particles with certain characteristics can act by pinning grain boundaries, inhibiting grain growth or inducing precipitated phase formation, such as AF (acicular ferrite), thus refining the grain and improving the microstructure^4,10–14^.

Extensive investigations have focused on particle-assisted microstructure control^1,15,16^. A. Mitchell *et al*. pointed out that the particles with distance larger than 10 μm and diameter smaller than 1 μm have no impact on macro-performance of metallic products^17^. The yield strength and tensile strength would increase remarkably for steels with particles less than 0.3 µm^18^. The fine MgO-containing particles were found to have a facilitating effect on the formation of equiaxed crystallization and refinement of microstructure^19–21^. Yang *et al*.^22^ reported that the proportion of AF progressively increased with increasing particle size from 1.0 to 1.8 μm and the ability of particles to induce AF was greatly reduced when the particle size reached 7.0 μm. Particles containing Ce with a size of about 4–7 μm can serve as heterogeneous nucleation sites for AF formation^23^. In spite of controversies on relation between particles with various compositions on the microstructure of metallic materials, some oxides, sulfides, nitrides and complex compounds (MnS, Ti_2_O_3_, TiN, VN, TiO·Al_2_O_3_·MnS, ect.) have been displayed promoting intra-granular ferrite nucleation or pinning grain boundaries^24–26^. The dispersed particles containing Ca or Mg are found to serve as heterogeneous nuclei for fine ferrite effectively due to the relatively weak affinity between individual particles and other characteristics^27^. Furthermore, Ca-containing alloy is commonly used to improve the continuous castability of liquid steel by modifying solid alumina particles into liquid calcium aluminates^28–30^. Yet, inappropriate addition of calcium can lead to the formation of calcium aluminate particles with large size. Wang *et al*.^31^ found that the stringer shaped particles longer than 150–350 μm in linepipe steel were deformed from calcium aluminate with 10–20 μm in cast slab which deteriorated the properties of low temperature toughness and hydrogen induced crack of steel. One of the key factors for decreasing the side effects of particles, or determining in the pining effect of particles or their ability on serving as cores of precipitated phase nucleation is the particle size.

The formation of particles starts with nucleation which plays an important role in determining the structure, shape and size distribution of the particles. Suito and Ohta *et al*.^32^ found that the initial size distribution of particles became narrow in the case of high nucleation rate, which they thought was facilitating to obtain fine particles. After that, the particles are deemed to grow and coarsen by the following steps: the diffusion of reactants to the oxide nuclei, Ostwald ripening^33^, collision and subsequent coagulation in liquid metal. Lindberg *et al*.^34^ reported that the time for attending the 90% of the equilibrium value of particle volume is 0.2 s. Suito and Ohta *et al*.^35^ investigated that the growth of particles by diffusion is very fast. In their study, Ostwald ripening dominates the growth of particles in deoxidation process under no fluid flow. However, in our previous study^36^, the experimental evidence for particles size distribution in Fe-O-Al-Ca melt corresponds to the theoretical results based on Ostwald ripening at early stage of deoxidation but not at later stage. Therefore, it is necessary to study the change of particle size distribution in liquid metal affected by the collision and subsequent coagulation. Collisions between particles and rapid diffusion in the liquid phase increase the number of large particles and enhance particle removal by floatation^37^. Extensive theoretical studies about the time-dependent particle size distribution and mathematical model have been reported based on collision-coalescence behavior due to turbulent collision^38^, Stokes collision^39–41^, Brownian collision^42^. Furthermore, attractive capillary force acted on particles has been also investigated with consideration on chemical compositions, size and distance between particles which is one mechanism for coagulation^43–46^. It can be concluded that the particle size distribution is effected by nucleation, growth, coagulation due to collision and attractive force, and floatation behavior of particles in liquid metal^47–52^. However, lots of researches are focused on the behavior of solid particles^32,34,53^ and there are limited researches on the coarsening of liquid particles in liquid metal. The nucleation and Ostwald ripening of liquid calcium aluminate particles in Fe-O-Al-Ca melt have been investigated in our previous study^36^. In current study, the coarsening mechanism of particles in Fe-O-Al-Ca melt was studied with the consideration of nucleation, coagulation due to collision and floating properties and verified by experimental data. This study will provide information to understand the relations between characteristics, behavior and coarsening of particles in Fe-O-Al-Ca melts, and will be helpful for predicting and controlling size of particles.

## Methods

Materials used in the present study and high temperature experimental processes are described detailedly in our previous study^36^. Characteristics of particles were detected by SEM at an accelerating voltage of 15 KV and the transformation of particle characteristic in three-dimensional from that in two-dimensional based on stereological analysis is the same with our previous study^36^.

Geometric standard deviation of particle size distribution ln *σ* values is calculated by Eq. (1)

1 $$ln{\sigma }={[\frac{{\sum }^{}{n}_{i}{(ln{r}_{i}-ln{r}_{geo})}^{2}}{{\sum }_{i=1}^{n}{n}_{i}}]}^{1/2}$$

Where *r*_*geo*_ is the geometric mean radius of particle given by (*r*_1_· *r*_2_· *r*_3_ …… *r*_*n*_)^1/n^. The ln *σ* values are obtained from Eq. (1) using the values for the size and number density of particles which were measured in the deoxidation experiments.

**Figure 1:**
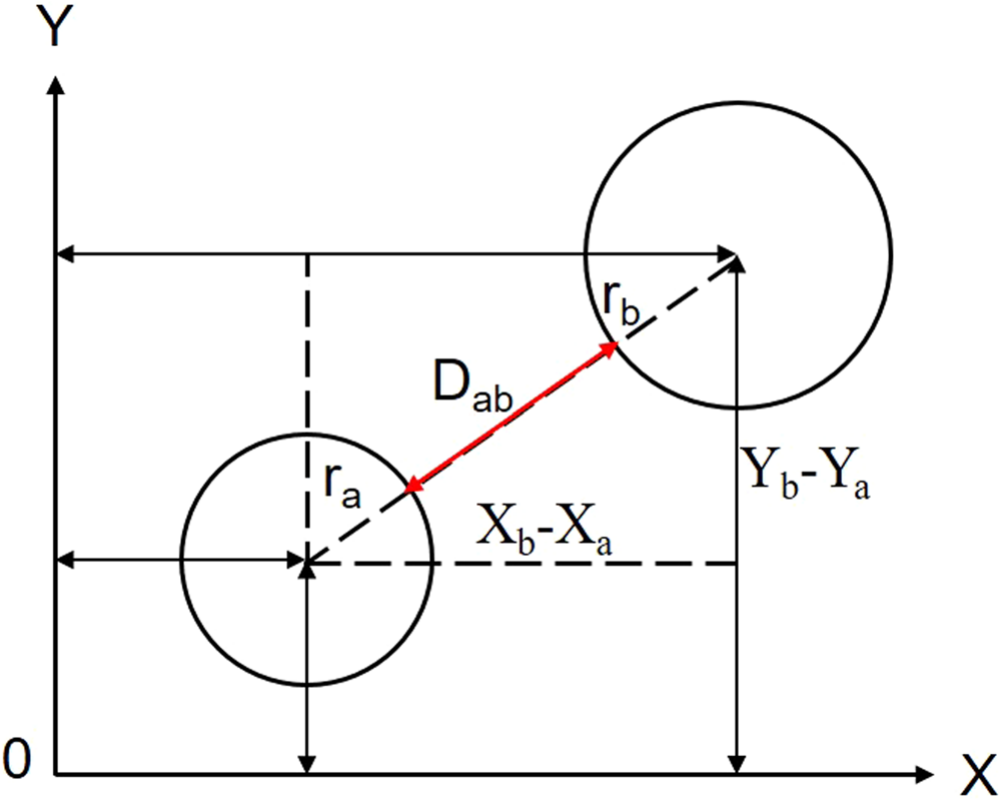
Illustration of inter-surface distance between particles.

The inter-surface distance *D*_*ab*_ between two particles can be obtained by measuring the central coordinates and radius of particles by Image-Proplus software and the illustration is shown in Fig. 1.

2 $${D}_{ab}=\sqrt{{({X}_{b}-{X}_{a})}^{2}+{({Y}_{b}-{Y}_{a})}^{2}}-{r}_{a}-{r}_{b}$$

Where *X*_*i*_ and *Y*_*i*_ are the central coordinates of particles in the cross section, *r*_*i*_ is the equivalent radius and *D*_*ab*_ is the inter-surface distance between two particles.

By calculating the inter-surface distances of certain particle with all others, the inter-surface distance between this certain particle with the nearest particle *D*_*mi*_ can be obtained by calculating the minimum value of *D*_*ab*_ as Eq. (3) and *D*_*mi*_ is defined as the inter-surface distance of a pair of adjacent particles in this paper. The average inter-surface distance of particles in certain region of sample *D*_*AV*_ is the arithmetic mean value of *D*_*mi*_ calculated as Eq. (4).

3 $${D}_{mi}=MIN({D}_{i1},{D}_{i2}\,\cdots \,{D}_{ik})$$

4 $${D}_{AV}=\frac{{\sum }_{i=1}^{k}{D}_{mi}}{k}$$

## Results

### *In-situ* observation of liquid particle behavior

**Figure 2:**
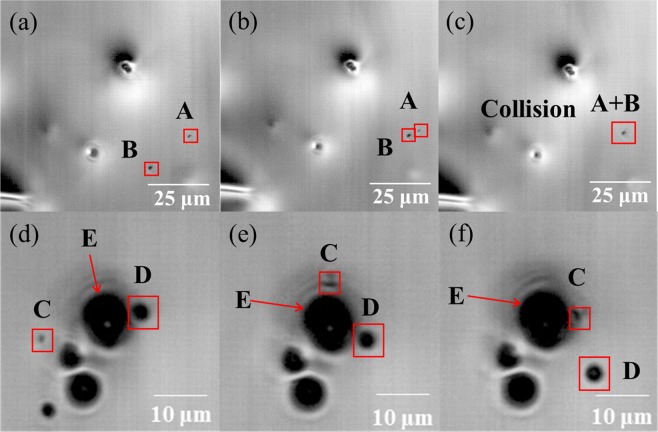
*In-situ* observation of calcium aluminate inclusions on the surface of Fe-O-Al-Ca melt by HT-CSLM. (**a**) Particles A and B at 0 s in field 1; (**b**) particles A and B at 0.22 s in field 1; (**c**) particles A and B at 0.66 s in field 1; (**d**) particles C, D and E at 0 s in filed 2; (**e**) particles C, D and E at 0.55 s in field 2; (**f**) particles C, D and E at 1.21 s in field 2.

The behavior of particles in Fe-O-Al-Ca melt was *in-situ* observed using high-temperature confocal scanning laser microscope (HT-CSLM) as shown in Fig. 2. Most aggregation and coagulation between liquid calcium aluminates were caused by collision as Fig. 2(a–c). The attraction force was hardly found between most liquid calcium aluminate particles at gas/molten steel interface, even at very small separation (particle C/D moved to particle E and then passed away) as shown in Fig. 2(d–f). The same phenomenon was also observed by Hongbin Yin^54^, in which they found that the liquid calcium aluminate particles could separate freely after getting in touch with each other at 1/6 seconds.

### Characteristics of particles

**Figure 3:**
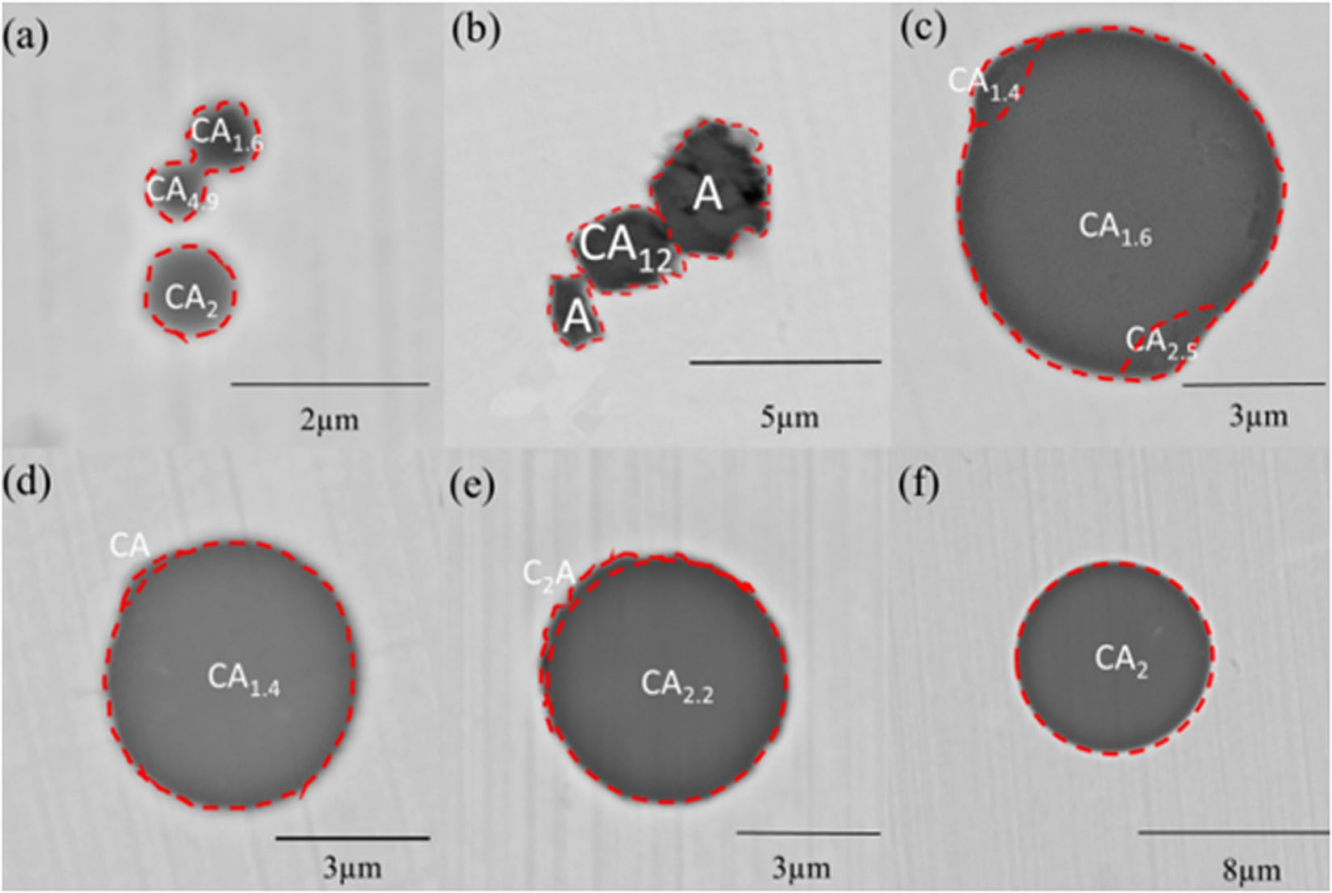
SEM-EDS analysis of typical calcium aluminate particles in Fe-O-Al-Ca melts after deoxidation at 1600 °C for 360 s.

Morphologies and compositions of particles in steels deoxidized by Al and Ca alloys SEM-EDS micrographs are displayed in Fig. 3. It can be seen that there were lots of calcium aluminate particles in collision and coagulation. Few particles with similar size were found jointed as Fig. 3(a). According to the low melting point diagram Ca-Al-S^55^, the particles with mole ratio of Al_2_O_3_ to CaO in the range of 0.15–1.5 are in liquid or partially liquid state which are regarded as “liquid particles” in this paper. Some solid particle merged with each other and formed into an irregular aggregate by high temperature sintering which was hard to deform and densify as Fig. 3(b). The coagulations between the liquid particles were observed as Fig. 3(c–e) and these aggregates seem susceptible to deform into spherical body. Such difference is thought to be attributed to the difference of inter-diffusion of composing elements and contact area, as the liquid particles are prone to spread on the surface of the other one^54^. Therefore, it can be concluded that the particles with large discrepancy in size tend to collide and merge, and the deformation as well as densification proceed easily for liquid calcium aluminates particles.

**Figure 4:**
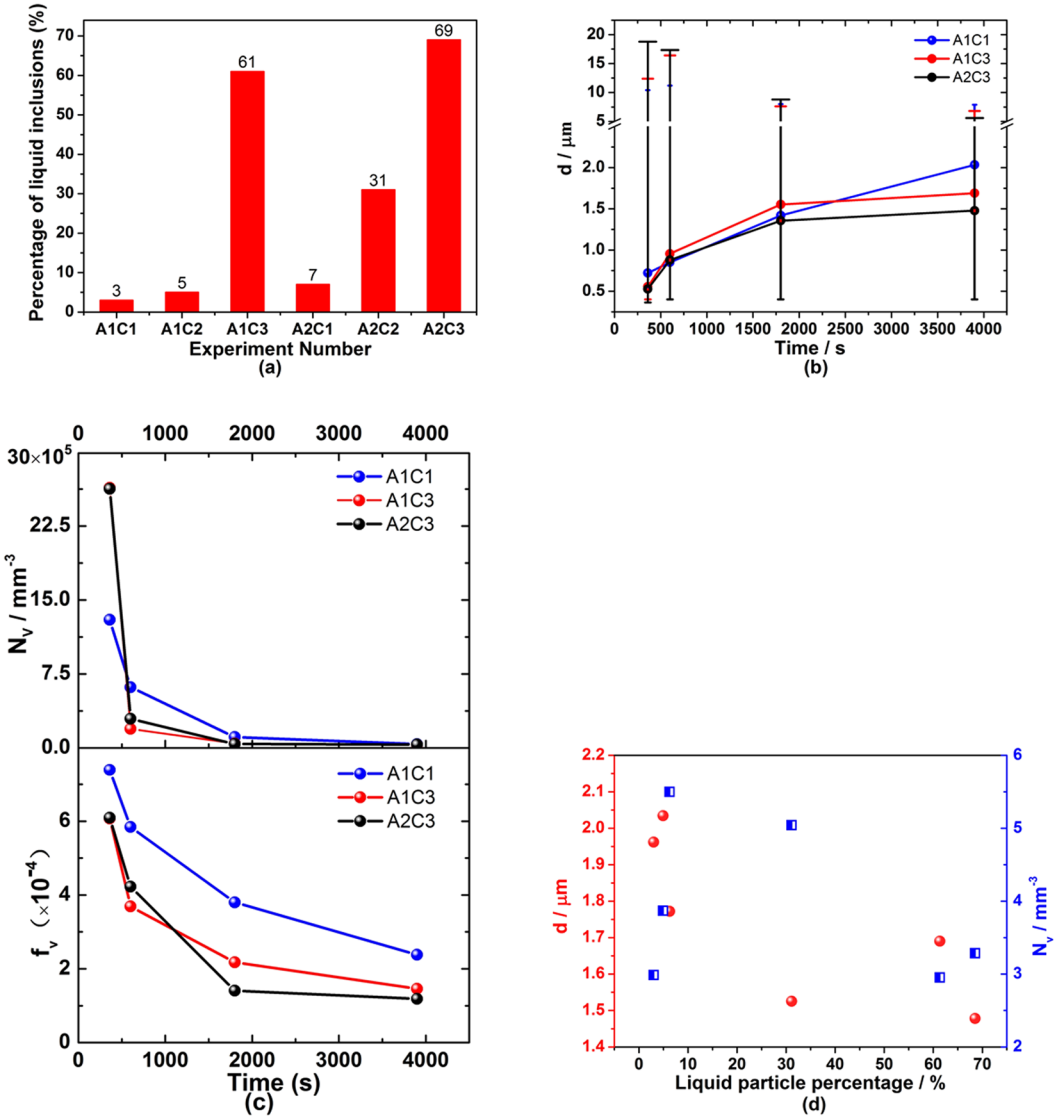
Percentage of liquid particles, diameter, number and volume fraction of particles in samples. (**a**) Percentage of liquid particles in each sample is observed by SEM-EDS in samples after deoxidation for 3900 s and it is calculated based on Ca-Al-S phase diagram^55^; (**b**) effect of holding time on particle diameter in three-dimensional based on stereological analysis and the error bars represent the maximum and minimum values of particle size; (**c**) effect of holding time on number and volume fraction of particles in three-dimensional; (**d**) change of average diameter and number density of particles in three-dimensional with liquid particle percentage, and the red circle and blue square represent average diameter and number density of particles, respectively.

In order to study the effect of liquid particle on their characteristics, the percentage of liquid particles in Fe-O-Al-Ca melts is illustrated in Fig. 4(a) (C1/2/3 represents that initial adding amount of Ca is 0.25%/0.4%/ 0.78%; A1/2 represents that initial adding amount of Al is 0.05%/0.25%). The experimental condition and chemical compositions of samples were depicted in previous study^36^. Liquid particle percentage increased with increasing amount of calcium addition in melts. The steels with high calcium addition ([%Ca] = 0.78) after deoxidation 3900 s have an extremely higher percentage of liquid particles than those with low calcium addition ([%Ca] = 0.25, 0.4).

A few hundreds of particles were observed by SEM-EDS in each sample and the planar particle size distribution was transformed into the size, number density and volume fraction of particles in three-dimensional based on stereological analysis. The average size of particles increased, and their number density and volume fraction decreased significantly with holding time as illustrated in Fig. 4(b,c). The error bars in Fig. 4(b) indicates that the particles with 18 μm formed in experiment A2C3 ([% Al]_i_ = 0.25 and [% Ca]_i_ = 0.78) during the first 360 s of deoxidation and subsequently those with 16 μm formed in experiment A1C3 ([% Al]_i_ = 0.05 and [% Ca]_i_ = 0.78), but no particles with size larger than 12 μm were observed in experiment A1C1 ([% Al]_i_ = 0.05 and [% Ca]_i_ = 0.25). The size of the largest particle in each experiment was larger in the steel with higher Ca addition during the first 360 s of deoxidation, and decreased with holding time due to the rapid floatation of large particle^56^ which was explained in DISCUSSION part. The change of number density and volume fraction for calcium aluminates in Fig. 4(c) suggests that the ascending velocity of particles in the steel containing more liquid particles (A1C3 and A2C3) was larger than that containing more solid particles (A1C1) at early stage due to the relatively larger size and fractal dimension of liquid particles in the steels with high calcium (In spite of larger density for solid calcium aluminate particles, the liquid particles in the steel containing high calcium were larger in size and fractal dimension relatively at the initiation of deoxidation, which accelerated the floatation of these liquid particles). It is reported that the ascending velocity of the condensed particles with fractional characteristic was smaller than that of isometric three dimension spherical particles^43^, and it decreased with the decreasing value of D_f_ (fractal dimension)^57^. Based on the expression of D_f_ by Lech Gmachowski^58^, the liquid aggregates with spherical shape have larger D_f_ than the irregular solid aggregates.

With the rapid rise of large particles, the average size of particles increased slightly and their number density decreased slowly after deoxidation for 1800 s. Furthermore, the change of characteristics for particles in the steels containing high calcium (with low number density of particle) was smaller than that in the case with low calcium (with high number density of liquid particle) at later stage of deoxidation. It is thought to be caused by the difference of collision rate affected by the number density which can be verified in DISCUSSION part. Therefore, as illustrated in Fig. 4(d), the average diameter tended to decrease with an increased percentage of liquid particles due to the rapid rise of particles in high Ca containing steel at early stage of deoxidation and less collision at later stage. The number density of particles changed irregularly which is affected by their aggregation and floatation behaviors.

### Spreading of particle size

**Figure 5:**
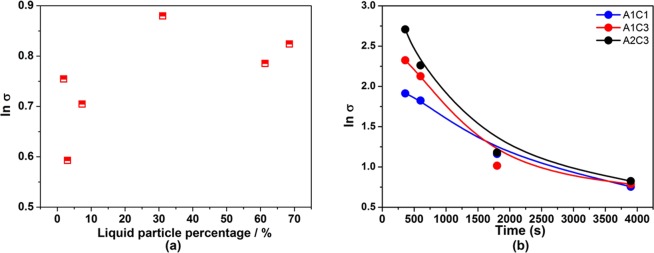
Geometric standard deviation of particles as a function of liquid paricle percentage and holding time in Fe-O-Al-Ca melts. (**a**) ln σ values of particle as a function of liquid particle percentage in Fe-O-Al-Ca melts after deoxidation at 1600 °C for 3900 s; (**b**) ln σ values of particle as a function of holding time in experiment A1C1 ([% Al]_i_ = 0.05 and [% Ca]_i_ = 0.25), A1C3 ([% Al]_i_ = 0.05 and [% Ca]_i_ = 0.78) and A2C3 ([% Al]_i_ = 0.25 and [% Ca]_i_ = 0.78).

The geometric standard deviation of particle size distribution ln *σ* value which represents the spreading of particle size distribution in each experiment was measured in the deoxidation experiments. It is illustrated in Fig. 5(a) that the ln *σ* values had an increasing trend with the increase of liquid particle percentage after deoxidation for 3900 s and they decreased with time elapsed. As the spreading of a size distribution becomes narrower with a decrease in ln *σ*, it means that discrepancy in particle size is greater in the case with more liquid particles. Ohta *et al*.^35^ reported that ln *σ* values were dependent on the nucleation rate in the early stage. It can be seen that in Fig. 5(b) that the ln *σ* values increased in the order of Exp. A1C1 < Exp.A1C3 < Exp.A2C3 at the first 600 s of deoxidation process, in which the theoretical nucleation rates ln I were 484, 313, and 309, respectively (as reported in our previous study^36^). This result is in agreement with the conclusion that in the case of low nucleation rate, the particle size distribution becomes broader^35^. The ln *σ* values of calcium aluminate particle at 3900 s changed non-monotonically with the increase of liquid particle percentage due to the hereditary of particle size distribution from early stage of deoxidation and the change of particle number density.

### Inter-surface distance between particles

**Figure 6:**
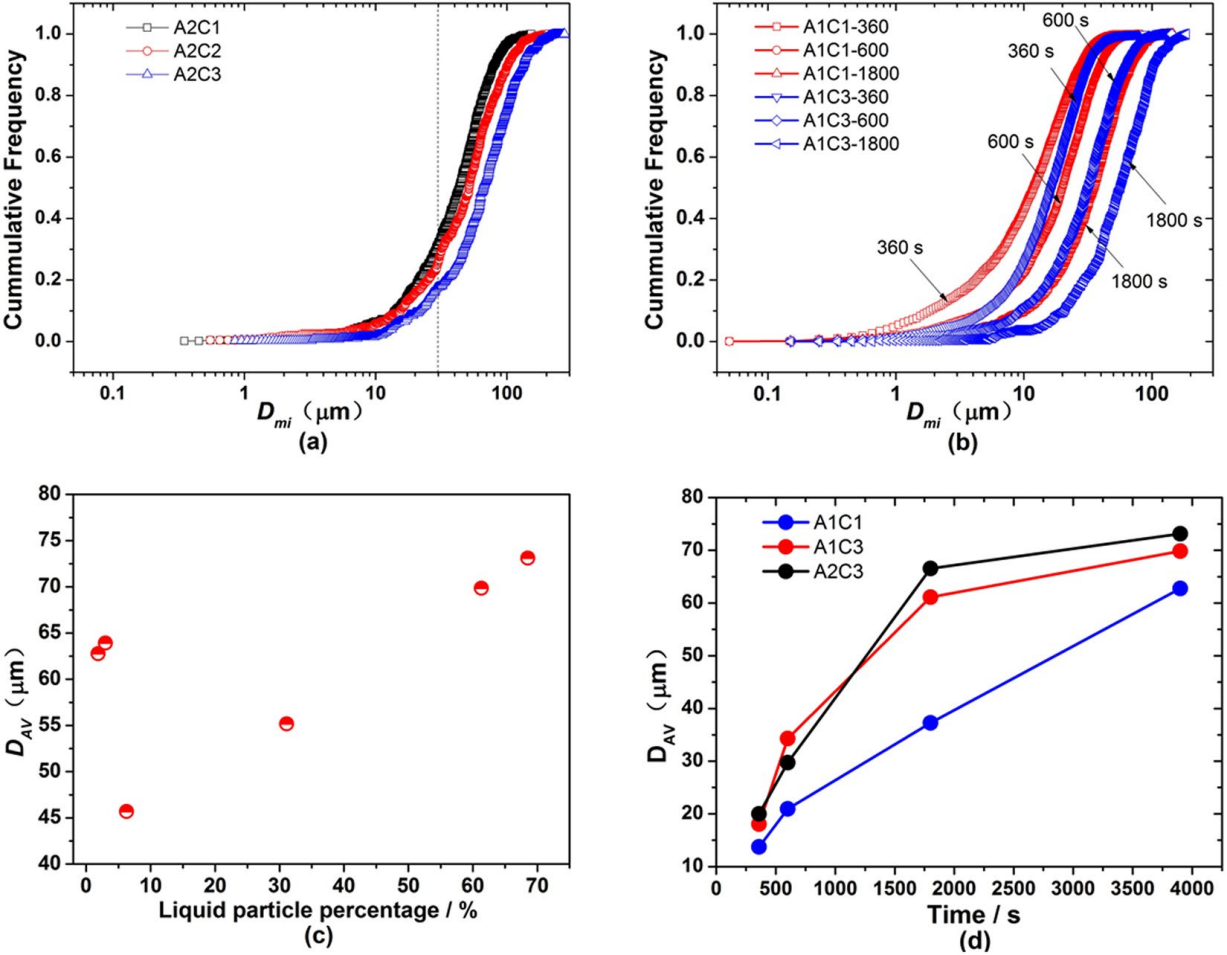
Inter-surface distance of particles in Fe-O-Al-Ca melts. (**a**) Cummulative frequency of inter-surface distance of adjacent particles D_mi_ in steels with different amount of Ca addition after deoxidaiton 3900 s and D_mi_ is obtained based on Eqs (7 and 8). (**b**) Cummulative frequency of D_mi_ in steels with holding time in experiments A1C1 ([% Al]_i_ = 0.05 and [% Ca]_i_ = 0.25) and A1C3 ([% Al]_i_ = 0.05 and [% Ca]_i_ = 0.78). (**c**) Average distance of particles D_AV_ as a function of liquid particle percentage in Fe-O-Al-Ca melts after deoxidation for 3900 s and D_AV_ is obtained based on Eqs (7–9). (**d**) D_AV_ values of particles as a function of holding time in experiments A1C1 ([% Al]_i_ = 0.05 and [% Ca]_i_ = 0.25), A1C3 ([% Al]_i_ = 0.05 and [% Ca]_i_ = 0.78) and A2C3 ([% Al]_i_ = 0.25 and [% Ca]_i_ = 0.78).

The cumulative frequency curves of *D*_*mi*_ (inter-surface distance of adjacent particles) in Fig. 6(a) change little, and the particles with inter-surface distance of 30–100 μm were in larger proportion. The curves in Fig. 6(a) move toward right with increasing addition of calcium at 3900 s which means that the particles were in larger inter-surface distance with more calcium addition. Figure 6(b) shows that the proportion of particles with close inter-surface distance (<10 μm) accounted for 40%, 25% after deoxidation for 360 s in experiments A1C1 and A1C3, and it reduced to 20% and 8% after deoxidation for 600 s. The inter-surface distance between farthest particles increased with time elapsed. The average inter-surface distances of particles in Fig. 6(c,d) show that the D_*AV*_ values (average inter-surface distance of particles in certain region of sample) decreased with the increasing liquid particle percentage when it was larger than 5%, and they increased with time elapsed. It is noteworthy that the change rule of particle inter-surface distance is contracted with that of particle number density, indicating that the larger the number density of particle is, the closer the particles are.

### Distribution of particles

**Figure 7:**
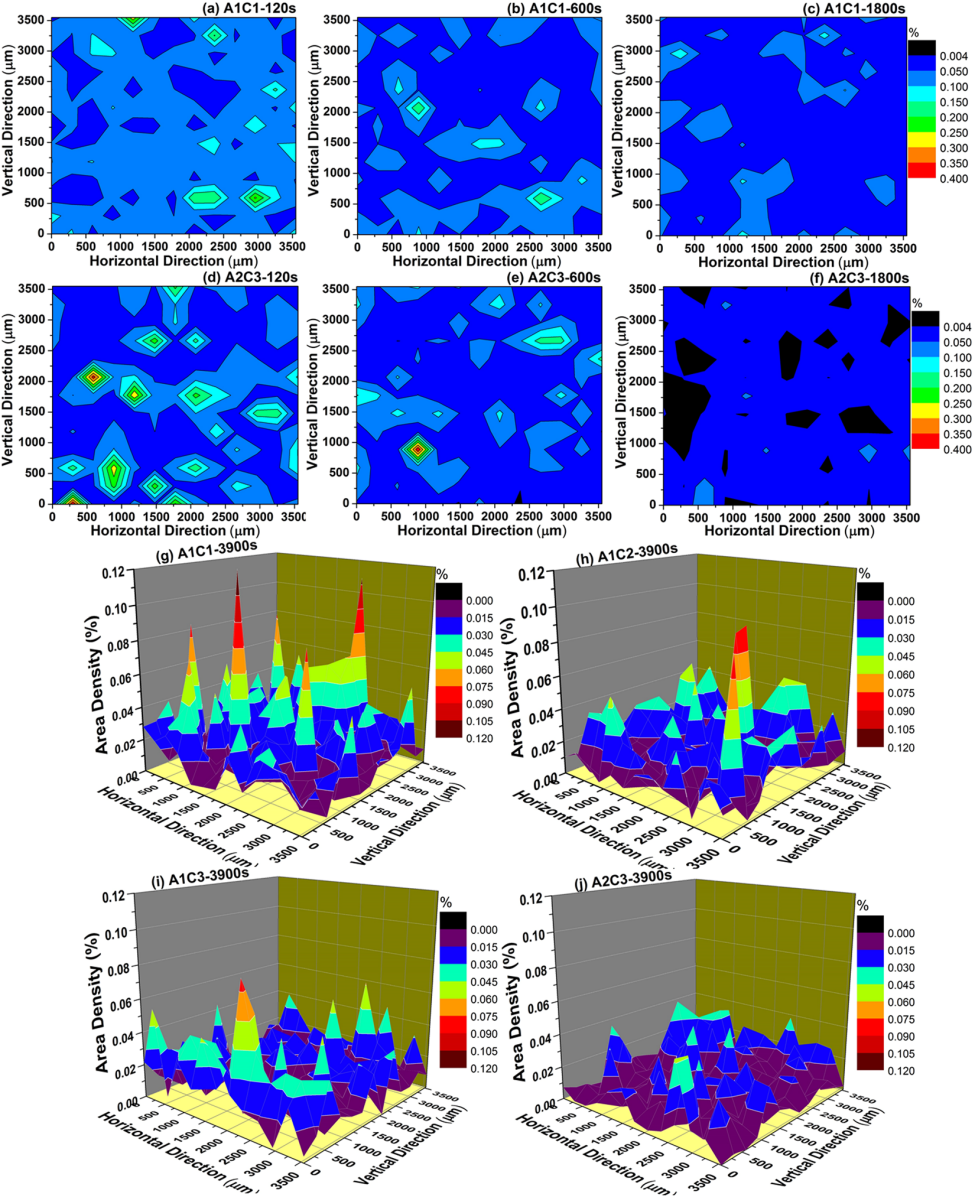
Distribution of area density for particles on the cross section in Fe-O-Al-Ca melts, counting from SEM images.

The distribution of area density for particles in Fe-O-Al-Ca melts as a function of holding time and amount of Al and Ca addition is displayed in Fig. 7. The segregation of particles were more serious in Fe-O-Al-Ca melts containing high Ca at early stage of deoxidation due to high number density which enhanced the collision and coagulation of particles, resulting in larger particles. It can explain the phenomenon that the size of largest particle increased in the order of A2C3 > A1C3 > A1C1 as shown in Fig. 4(b). With time elapsed, the area density for particles in steel decreases due to the floatation of particles. As the floatation of particle in the liquid steel following stokes behavior^56^, the particles with larger size have higher ascending velocity, and thus, in the high Ca containing steel, more particles with large size were removed after deoxidation for 1800s which resulted in lower area density for particles at later stage of deoxidation. It can also be verified in Fig. 7(g–j) and it indicates that the particles distribute more homogenously and their area density decreased with the increasing amount of Ca addition, resulting in relatively fine particle in melts with high Ca addition, corresponding to Fig. 4(d).

## Discussion

### Collision of particles

As result shows, the characteristics of particles in Fe-O-Al-Ca melts are dependent on their collision and coagulation behavior. The collision rate of particles in the liquid steel can be estimated as population balance model for collision^44,59^:

5 $$\frac{d{n}_{i}}{dt}=\frac{1}{2}\sum _{k=1}^{i=1}(1+{\delta }_{k,i-k}){\beta }_{k,i-k}{n}_{k}-{n}_{i}\sum _{k=1}^{{i}_{M}}(1+{\delta }_{i,k}){\beta }_{i,k}{n}_{k}$$

Where d*n*_i_/d*t* is collision rate of particles (mm^−3^·s^−1^), *n*_i_ is the number of size *i* particles per unit volume (mm^−3^) and *β*_*i*,*j*_ is the collision frequency between size *i* and size *j* particles (m^3^·s^−1^). δ_i,k_ is the Kronecker delta^60^, δ_i,k_ = 1 for i = k, and δ_i,k_ = 0 for i ≠ k. When i = 1, the Equation simplifies to

6 $$\frac{d{n}_{i}}{dt}=-{n}_{1}\sum _{k=1}^{{i}_{M}}(1+{\delta }_{1,k}){\beta }_{1,k}{n}_{k}$$

In this experiment, only Brownian collisions and Stokes collisions happened among the particles in Fe-O-Al-Ca melts, and it’s not necessary to consider the turbulent collisions without external stirring condition. Therefore, the collision frequency *β*_*i*,*j*_ between size *i* and size *j* particles can be estimated as:

7 $${\beta }_{ij}={\beta }_{ij}^{S}+{\beta }_{ij}^{B}$$

8 $${\beta }_{ij}^{S}=\frac{2g\pi ({\rho }_{Fe}-{\rho }_{MxOy})}{9\mu }({r}_{i}+{r}_{j})$$

9 $${\beta }_{ij}^{B}=\frac{2kT{({r}_{i}+{r}_{j})}^{2}}{3\mu {r}_{i}{r}_{j}}$$

Where *β*^S^_ij_ is Stokes collisions as a result of the difference in ascending velocity of particles and clusters in the liquid steel (m^3^·s^−1^), *β*^B^_ij_ is Brownian collisions as a result of random movements of particles in the melt (m^3^·s^−1^), and *μ* is the dynamic viscosity of steel (=0.006 kg/m·s).

**Figure 8:**
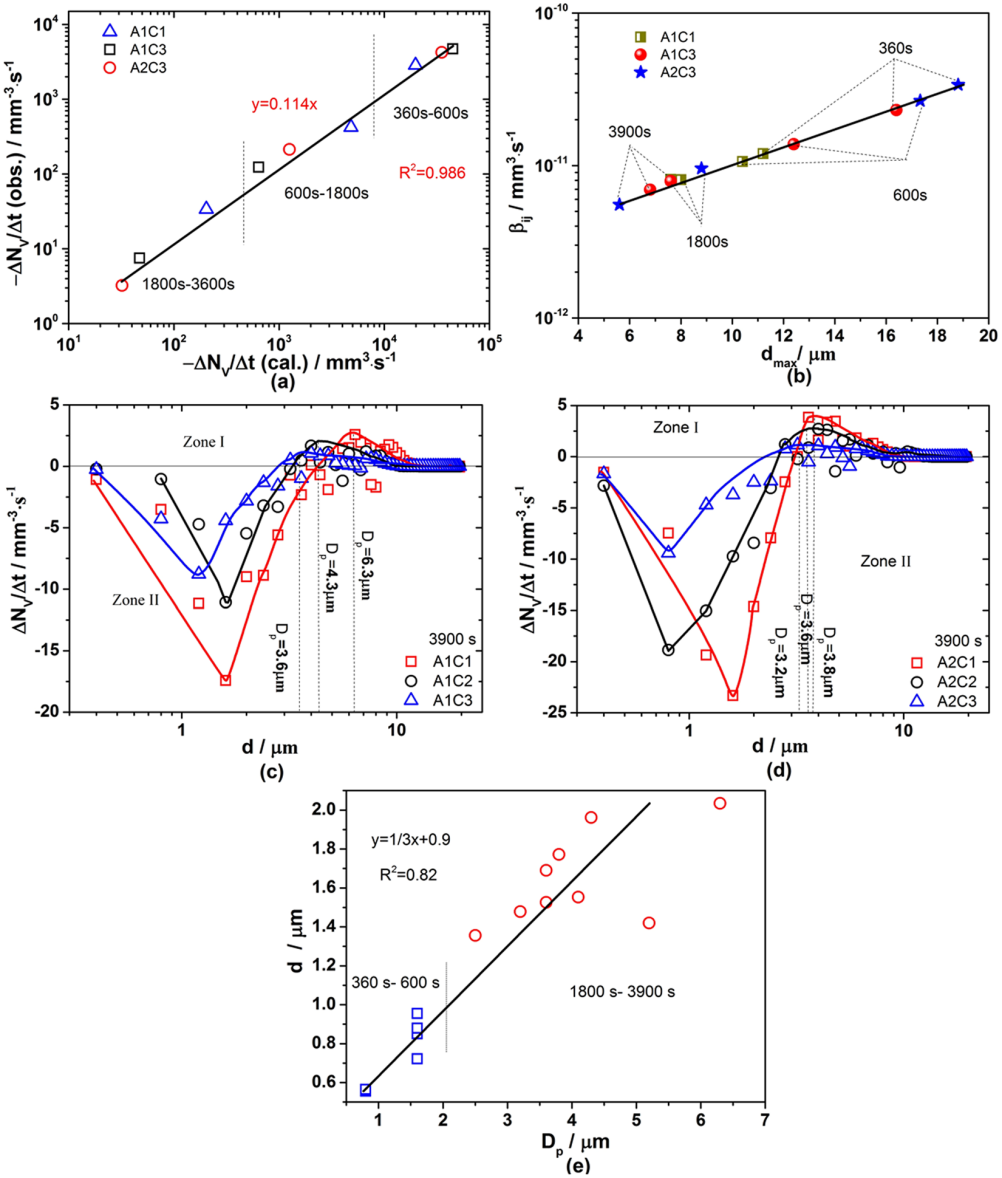
Collision rate of particles in Al-Ca deoxidation steel. (**a**) Experimental change rate of number density is obtained by measuring the total number of particles in Fe-O-Al-Ca melts with holding time and plotted against calculated collision rate of particles based on population balance model; (**b**) collision frequency for particles as a function of maximum size of particle in Fe-O-Al-Ca melts during deoxidation process based on Eq. (3); (**c**) Collision rate of particles with different size in melts with 0.05% Al addition after deoxidation for 3900 s; (**d**) Collision rate of particles with different size in melts with 0.25% Al addition after deoxidation for 3900 s, (**e**) Relationship between arithmetic mean diameter of particles with D_p_ value and D_p_ value is the size of particles corresponding to the peak value of curves in Fig. 6c,d.

The experimental change rate of particle number density (−ΔN/Δt) increases monotonically with collision rate of particles in Fig. 8(a). The observed values of −ΔN/Δt are about 1/9 of calculated collision rate which indicates that not all the particles will coagulation after collision. Compared with the total collision rate in the steel containing low calcium, it is higher in the case of high calcium during the first 600 s, while becomes lower at the later stage of deoxidation process. Moreover, the collision rate of particles decreases with time elapsed which is attributed to a decrease of number density. Figure 8(b) illustrates that the size of largest particle in each sample increases with an increased collision frequency *β*_*i*,*j*_ which decreases with time elapsed. It is verified that the collision behavior of particles affects their size significantly.

Figure 8(c,d) represents the collision rate of particles with different size at 3900 s. Zone I and zone II in Fig. 8(c,d) represent the particles with certain size increase and decrease, respectively. The particles with small size (<3 μm) decrease and those with large size (>3 μm) increase; the particle change rate of number density for particles, *i*.*e*. absolute value ΔN/Δt, increases with the increasing size of particles, and then decreases with further increase of particle size in both zone I and zone II. Comparing the curves in Fig. 8(c,d), it is found that the peak values of curves and the size of particles corresponding to those increase with the decrease of the valley values, which means that, the more the small particles reduce, the more the large particles form by collision and the bigger the produced particles with largest number density are. Furthermore, it is found that the size of particles corresponding to the peak value of curves, D_p_, decreases with the increase of Ca addition, which is the same with the changing trend of arithmetic mean diameter of particles observed in experiments. Hence, D_p_ is plotted with arithmetic mean diameter of particles in Fig. 8(e). It seems that the D_p_ values go up with an increase of the arithmetic mean diameter of particles linearly, especially at later stage of deoxidation process, while they change little with the increase of during the first 600 s of deoxidation process. It indicates that the collision between particles affects the coarsening of the particles at later stage of deoxidation but not early stage under the condition of no stirring, which is in agreement with the conclusion in previous study^36^.

### Influencing factors on collision of particles

**Figure 9:**
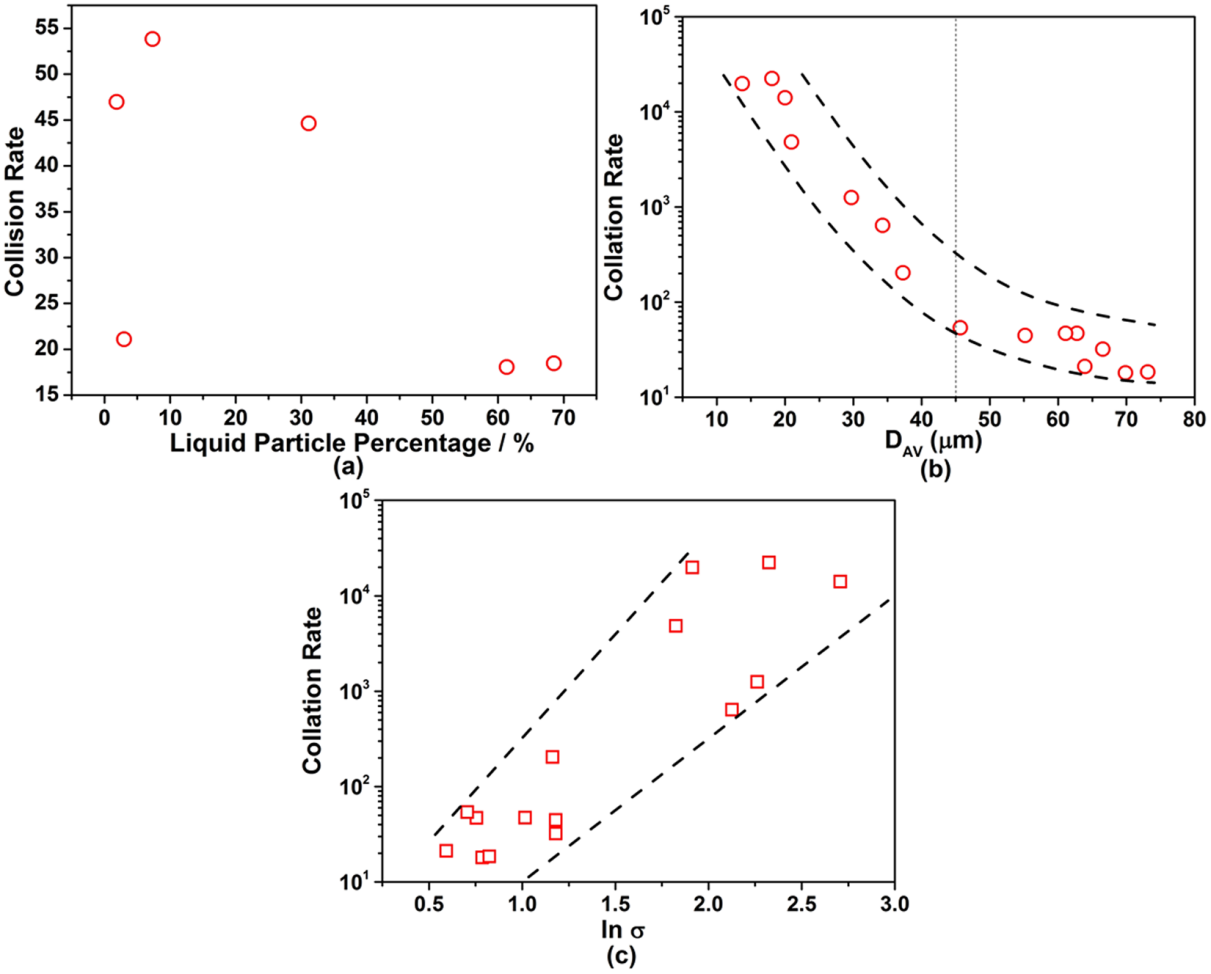
Change rule of collision rate for particles in Fe-O-Ca-Al melts. (**a**) Collision rate for particles as a function of liquid particle percentage; (**b**) Collision rate for particles as a function of average inter-surface distance between particles; (**c**) Collision rate for particles as a function of geometric standard deviation of particle size distribution.

The effect of liquid particle percentage, average inter-surface distance between particles and ln σ values on the collision rate for particles are summarized in Fig. 8. The changing trend of collision rate with liquid particle percentage is the same with number density in Fig. 4(d) and contrary to inter-suface distance in Fig. 6(c), indicating that the collision rate is mainly affected by particle number density and the distance of particles; as particle number density increases, the inter-surface distance between particles decreases and then the collision rate increases. Figure 9(b) shows an obvious decrease in collision rate with increasing D_AV_ values from 10 to 45 μm; however, with further increasing D_AV_ values, the collision rate exhibits a slight decrease. The change of collision rate with ln *σ* values in Fig. 8c shows that the collision rate increases with the increase of ln *σ* values, which means that the collision rate of particles with broad spreading of size distribution is higher than that with narrow spreading of size distribution.

### Coarsening mechanism of particles

**Figure 10:**
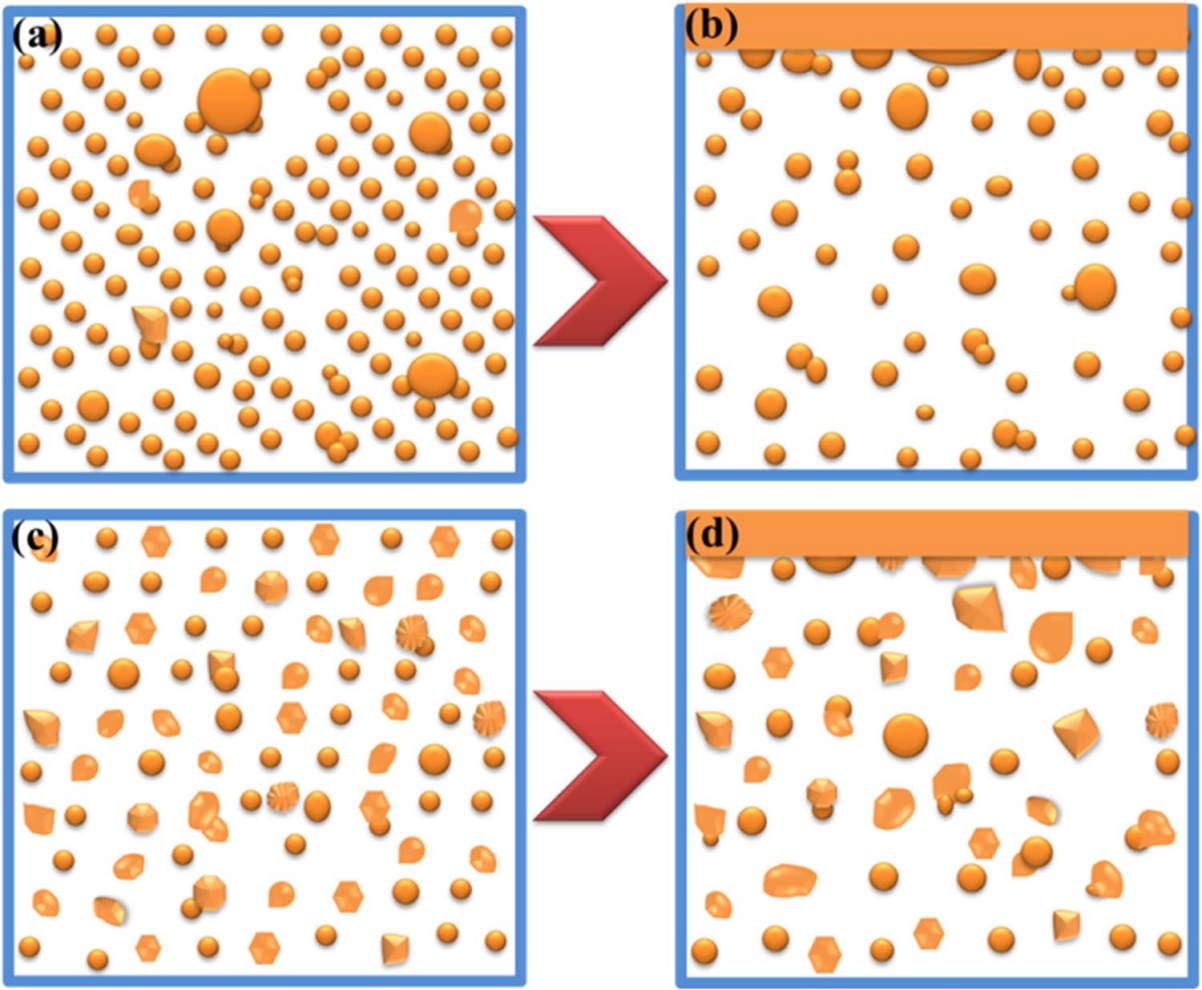
Schematic diagram of particle coarsening in Fe-O-Al-Ca. (**a**) Characteristics of particles for melts containing high Ca at early stage of deoxidation; (**b**) Characteristics of particles for melts containing high Ca at later stage of deoxidation; (**c**) Characteristics of particles for melts containing low Ca at early stage of deoxidation; (**d**) Characteristics of particles for melts containing low Ca at later stage of deoxidation.

The coarsening mechanism of particles in Fe-O-Al-Ca melts can be summarized in Fig. 10. The collision and coagulation of particles start after their nucleation and continue during whole deoxidation process. In the case of low nucleation rate and small inter-surface distance, the collision and coagulation tend to occur more easily, and hence, the maximum size of particle is larger in early stage of deoxidation as shown in Fig. 10(a) which is in agreement with the experimental result that the size of largest particle is larger in the steel containing higher Ca. Nevertheless, it is verified in our previous study^36^ that the average size of particles is mainly dependent on the Ostwald growth. As the rapid rise of liquid particles with large size and fractal dimension in Fig. 10, the inter-surface distance between particles in high Ca-containing melts becomes large and it is larger than that in low Ca-containing melts as in Fig. 10(b–d). With the consumption of Ca, Al and O, the coarsening of particles is mainly affected by their collision, but not Ostwald growth at later stage of deoxidation process. Therefore, the size of particles decreased with an increase of Ca addition after deoxidation for 3900 s.

## Conclusion

The behavior and characteristics of particles in the Fe-O-Al-Ca melts under the condition of no external stirring at 1600 °C was investigated using HT-CSLM, SEM-EDS detection. Most aggregation and coagulation observed between calcium aluminate particles were caused by collision. The characteristics of particles in three-dimensional, *i*.*e*. size, number density, volume fraction, spreading of particle size, inter-surface distance and distribution based on stereological analysis indicate that their coarsening is not only dependent on Ostwald growth as studied in previous study, but also collision and coagulation, and floatation. The collision of particles affects the maximum size of particle during whole deoxidation process and dominates the coarsening of particles at later stage of deoxidation. The calculated result based on population balance model indicates that the collision rate of particles increases with an increase of their number density, *i*.*e*. decrease of inter-surface distance, and it is high in the case for particles with narrow spreading of size distribution which is affected by nucleation rate. The particles with relatively larger size and fractal dimension have higher ascending velocity, resulting more fine particles with large inter-surface and low collision rate left in the melts. This mechanism can be used to explain that the collision, coarsening behavior and characteristic change of particles in melts with different amounts of Ca addition.

## Data Availability

The data that support the findings of this study are available from Linzhu Wang upon reasonable request.
